# Association of Clonal Hematopoiesis in DNA Repair Genes With Prostate Cancer Plasma Cell-free DNA Testing Interference

**DOI:** 10.1001/jamaoncol.2020.5161

**Published:** 2020-11-05

**Authors:** Kendal Jensen, Eric Q. Konnick, Michael T. Schweizer, Alexandra O. Sokolova, Petros Grivas, Heather H. Cheng, Nola M. Klemfuss, Mallory Beightol, Evan Y. Yu, Peter S. Nelson, Bruce Montgomery, Colin C. Pritchard

**Affiliations:** 1 Department of Laboratory Medicine, University of Washington, Seattle; 2 Department of Medicine, Division of Medical Oncology, University of Washington, Seattle; 3 Brotman Baty Institute for Precision Medicine, Seattle, Washington; 4 Clinical Research Division, Fred Hutchinson Cancer Research Center, Seattle, Washington; 5 Human Biology Division, Fred Hutchinson Cancer Research Center, Seattle, Washington

## Abstract

**Importance:**

Cell-free DNA (cfDNA) testing is increasingly used in the treatment of patients with advanced prostate cancer. Clonal hematopoiesis of indeterminate potential (CHIP) can interfere with cfDNA testing and cause incorrect interpretation of results. There is an urgent need to better understand this problem following recent US Food and Drug Administration approval of poly(ADP) ribose polymerase inhibitors (PARPi) for metastatic prostate cancer based on variants in DNA repair genes that can be affected by CHIP.

**Objective:**

To determine the prevalence of clinically relevant CHIP interference in prostate cancer cfDNA testing.

**Design, Setting, and Participants:**

We report a case series of 69 patients with advanced prostate cancer (metastatic disease or with rising PSA following localized therapy) who had cfDNA variant testing with a large panel cancer next generation sequencing assay (UW-OncoPlexCT). To determine the source of variants in plasma, we tested paired cfDNA and whole blood control samples. The study was carried out in an academic medical center system reference laboratory.

**Main Outcomes and Measures:**

Prevalence and gene spectrum of CHIP interference in patients with prostate cancer undergoing cfDNA testing.

**Results:**

We detected CHIP variants at 2% or more variant fraction in cfDNA from 13 of 69 men with prostate cancer (19%; 95% CI, 10%-30%). Seven men (10%; 95% CI, 4%-20%) had CHIP variants in DNA repair genes used to determine PARPi candidacy, including *ATM* (n = 5), *BRCA2* (n = 1), and *CHEK2* (n = 1). Overall, CHIP variants accounted for almost half of the somatic DNA repair gene variants detected. Participant CHIP variants were exponentially correlated with older age (*R*^2^ = 0.82). CHIP interference variants could be distinguished from prostate cancer variants using a paired whole-blood control.

**Conclusions and Relevance:**

In this case series, approximately 10% of men with advanced prostate cancer had CHIP interference in plasma cfDNA in DNA repair genes that are used for eligibility of PARPi therapy, most frequently in *ATM*. Clinical cfDNA testing should include a paired whole-blood control to exclude CHIP variants and avoid misdiagnosis.

## Key Points

**Question:**

How often are cell-free DNA (cfDNA) studies in prostate cancer confounded by clonal hematopoiesis (CHIP) variants in genes used for poly(ADP) ribose polymerase inhibitor (PARPi) eligibility?

**Findings:**

In this case series study of 69 men with advanced prostate cancer, 7 (10%) had CHIP variants in genes used for US Food and Drug Administration-approved indications of PARPi treatment, most frequently in *ATM*.

**Meaning:**

Men with prostate cancer are at high risk of being misdiagnosed as being eligible for PARPi therapy using current cfDNA tests; assays should use a whole-blood control sample to distinguish CHIP variants from prostate cancer.

## Teaser

This case series study examines the prevalence of clonal hematopoiesis and its potential interference in prostate cancer cell-free DNA testing.

## Introduction

Cell-free DNA (cfDNA) variant analysis is used to guide treatment decisions for men with metastatic prostate cancer (mPC) and to enroll patients on clinical trials.^1^ Two poly(ADP) ribose polymerase inhibitors (PARPi) were recently granted US Food and Drug Administration (FDA) approval for use in selected patients with mPC based on DNA repair gene status: rucaparib for patients with *BRCA1* or *BRCA2* variants and olaparib for patients with *ATM, BRCA1, BRCA2, BARD1, BRIP1, CDK12, CHEK1, CHEK2, FANCL, PALB2, RAD51B, RAD51C, RAD51D* or *RAD51L* variants.^2^ Following these biomarker-guided approvals we expect cfDNA testing will sharply increase for patients with mPC because it offers the convenience and simplicity of testing on a blood sample in the advanced disease setting.^1,3,4^ Thus, there is an urgent need to understand cfDNA testing performance and sources of test interferences.

Clonal hematopoiesis of indeterminate potential (CHIP) is a known confounder of cfDNA testing.^5,6^ Clonal hematopoiesis of indeterminate potential variants are detected in both plasma and whole blood, whereas prostate cancer variants are detected in plasma only. Yet most commercial labs perform cfDNA testing using a plasma-only approach that cannot reliably distinguish variants derived from prostate cancer vs those arising from CHIP. To improve cfDNA assay performance, we developed an approach (UW-OncoPlexCT) that simultaneously analyzes plasma and paired whole-blood control samples.^4^ Using this paired testing approach we sought to determine to what degree CHIP interferes with the results of prostate cancer cfDNA testing.

## Methods

We retrospectively reviewed cfDNA study results from 69 patients with advanced prostate cancer (metastatic disease or with rising PSA following localized therapy) sequenced by our Clinical Laboratory Improvement Amendments (CLIA)-certified and College of American Pathologists (CAP)-accredited clinical UW-OncoPlexCT protocol. Plasma cfDNA and a paired whole-blood control sample were tested in every patient.^4,7^ We defined CHIP interference as a pathogenic variant with variant allele fractions (VAFs) of at least 2% in both the whole blood and plasma. Germline variants were distinguished from CHIP clones by tumor sequencing. Sequencing data analysis and variant interpretation were performed by an expert molecular pathologist (C.C.P.). All data were manually reviewed in the integrated genomics viewer (IGV) to exclude sequencing artifacts. Data were generated and preprocessed by the University of Washington NGS Laboratory and Analytics group. This study was performed in accordance with the Declaration of Helsinki guidelines and approved by the University of Washington/Fred Hutchinson Cancer Consortium institutional review board and all patients provided written informed consent.

## Results

We detected CHIP interference clones at least 2% variant fraction in 13 of 69 patients (19%; 95% CI, 10%-30%). Seven patients (10%; 95% CI, 4%-20%) had CHIP variants in DNA repair genes that are used for PARPi selection (*ATM* n = 5, *BRCA2*, n = 1 and *CHEK2*, n = *1*) (Figure) (Table). The 6 remaining patients had CHIP interference in genes frequently impacted by CHIP: *ASXL1*, *DNMT3A*, *PTEN*, *TET2*, and *TP53* (Figure) (eFigure in the Supplement).^8,9^

**Figure.**
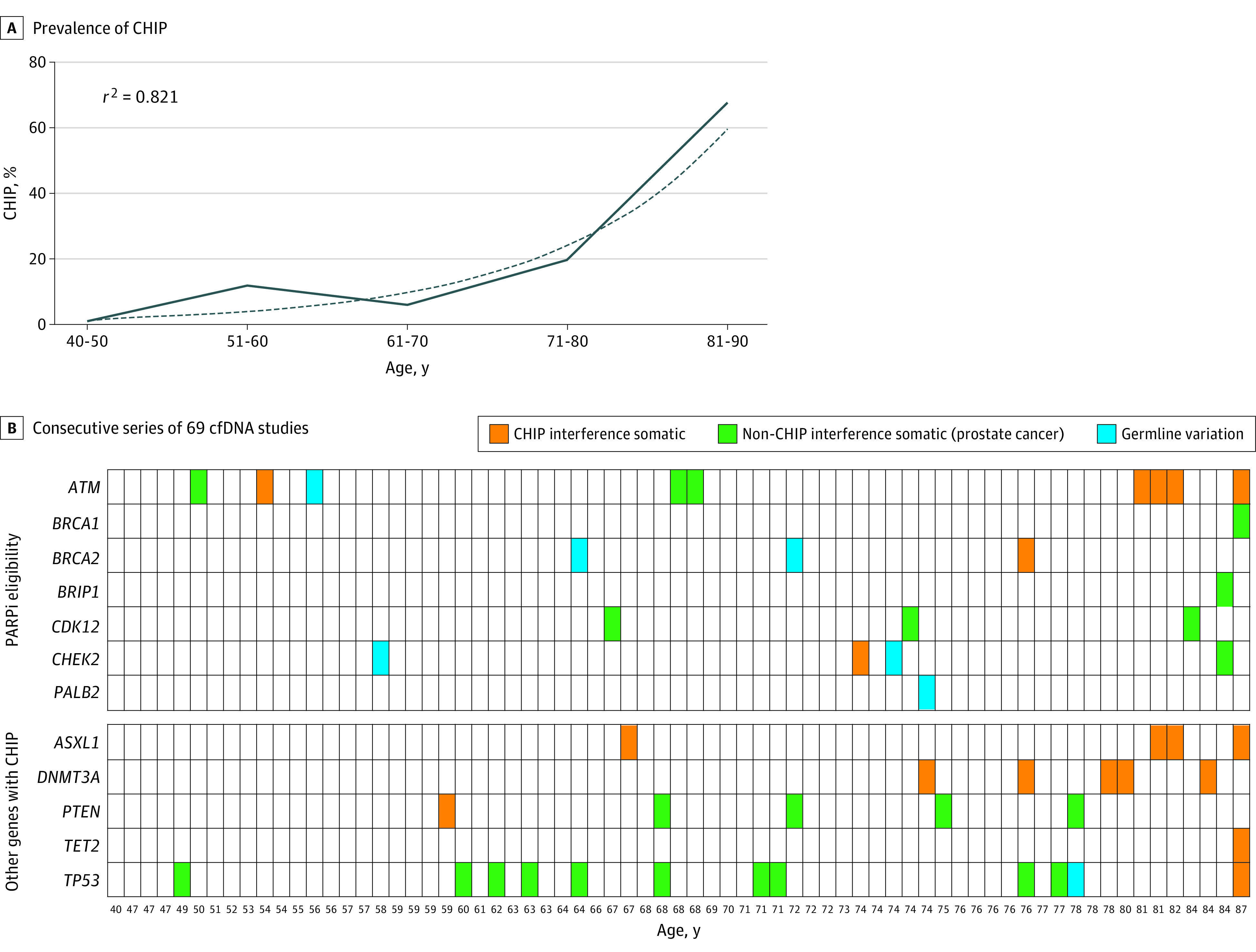
Source of Variants Detected in Prostate Cancer cfDNA Studies A, The prevalence of CHIP variants increased with age. CHIP was particularly prevalent (71%) in the 81 to 90 year age range. B, Consecutive series of 69 cfDNA studies. The DNA repair genes associated with PARPi eligibility are depicted along with other genes in which CHIP was detected. Each column represents 1 unique patient sorted by age. Variants detected in plasma are color coded by source, red indicates CHIP interference, somatic; green indicates non-CHIP, somatic (prostate cancer); yellow indicates germline. cfDNA indicates cell-free DNA; CHIP, clonal hematopoiesis of indeterminate potential; PARPi, poly(ADP) ribose polymerase inhibitor.

**Table.** CHIP Clones Detected in DNA Repair Genes Used for PARPi Eligibility

Age, y	Gene^a^	CHIP Variant(s)	VAF cfDNA	VAF blood control	Notes
81	*ATM*	p.R3008C, p.E3007D	16%; 5%	16%; 5%	CHIP hotspot, reported by outside lab in bone marrow
54	ATM	p.S305*	2%	3%	
82	*ATM*	p.G2891D	12%	13%	Kinase domain
81	*ATM*	c.2921 + 1G>A	78%	65%	Not germline based on tumor testing
87	*ATM*	p.L2492R	7%	9%	CHIP hotspot
76	*BRCA2*	p.T3310Nfs*17	3%	3%	Reported by outside lab, recommending PARPi
74	*CHEK2*	p.P426H	19%	18%	Kinase domain

Abbreviations: CHIP, clonal hematopoiesis of indeterminate potential; VAF, variant allele fraction; PARPi, poly(ADP) ribose polymerase inhibitor.

^a^ *ATM* reference sequence: NM_000051.3, *BRCA2* reference sequence NM_000059.3; *CHEK2* reference sequence: NM_007194.3.

We observed that CHIP interference correlated exponentially with increasing age (*R*^2^ = 0.82). We detected CHIP in 0% (0/6) of men aged 40 to 50 years, 12.5% (2/16) of men aged 51 to 60 years, 6.3% (1/16) of men aged 61 to 70 years, 20.8% (5/24) of men aged 71 to 80 years, and 71% (5/7) of men aged 81 to 90 years (Figure, A).

In 20 patients with advanced prostate cancer, we detected a total of 23 pathogenic variants in DNA repair gene variants used for selection of PARPi therapy, from the following source(s): CHIP interference somatic (n = 8, 1 patient had 2), non-CHIP somatic (n = 9), germline (n = 6) (Figure, B). We considered germline variants and non-CHIP somatic variants as true positives (n = 15) and CHIP interference as false positives (n = 8). Restricting the assay to a plasma-only analysis, only 65% of DNA repair gene variants detected were true positives (15/23). When incorporating a paired whole-blood control to remove CHIP interference, all DNA repair gene variants were true positives (15/15, 100%).

The patient with *BRCA2* CHIP interference had cfDNA testing done in parallel by an outside commercial laboratory using a plasma-only assay, which was unknown to our laboratory at the time of testing. The *BRCA2* CHIP clone was clinically reported by the commercial lab with the recommendation to use PARPi therapy.

## Discussion

We found that a strikingly high proportion of DNA repair gene variants in the plasma of patients with advanced prostate cancer are attributable to CHIP. The CHIP variants were strongly correlated with increased age, and even higher than expected by age group. The high rate of CHIP may also be influenced by prior exposure to chemotherapy.^10,11^ We are concerned that CHIP interference is causing false-positive cfDNA biomarker assessments that may result in patient harm from inappropriate treatment, and delays in delivering alternative effective treatment options. Without performing a whole-blood control, 7 of 69 patients (10%) would have been misdiagnosed and incorrectly deemed eligible for PARP-inhibitor therapy based on CHIP interference in plasma. In fact, 1 patient in this series had a *BRCA2* CHIP clone that had been previously reported by a commercial lab with the recommendation to use a PARPi. To mitigate these risks, cfDNA results should be compared to results from whole-blood control or tumor tissue.^12^

Challenges of accurate cfDNA testing are beginning to be described. A recent report^13^ highlighted inaccuracies of commercial laboratory cfDNA testing in patients with prostate cancer. In that report, cfDNA samples from 40 patients were sent to 2 separate CLIA-certified laboratories and only 9 of 40 (23%) demonstrated congruence (complete or partial) of positive findings.^13^ The consistent findings included *ATM* and *TP53* variants in patients with low PSA at the time of blood draw, raising suspicion that these may be CHIP clones. The CHIP interference in cfDNA testing has also been reported in other cancer types. In renal-cell carcinoma (RCC), for example, CHIP was found to affect cfDNA results in 43% of patients.^14^

Overall, *ATM* accounted for the majority of clinically relevant CHIP interference in our series. The *ATM* gene has been described as a frequent CHIP clone in clinical cancer predisposition testing, along with *CHEK2* and *TP53*.^10^ We speculate that CHIP interference in cfDNA testing could be affecting results of PARPi clinical studies of patients with metastatic prostate cancer. Trials allowing plasma-only cfDNA testing for enrollment may have included patients with false-positive results associated with CHIP in DNA repair genes, particularly in *ATM*.^15^ We speculate that this could be contributing to low PARPi response rates reported in patients with *ATM *variants, such as recently reported from the TRITON2 study.^15^

### Limitations

This study has several limitations including relatively small sample size, the retrospective nature of the study, and heterogeneity in patient populations and prior therapies.

## Conclusions

Findings of this study suggest that CHIP substantially interferes with plasma cfDNA testing in patients with advanced prostate cancer. There is a risk for widespread misdiagnosis and overtreatment of men with PARPi using currently available commercial cfDNA assays. We recommend that all cfDNA testing in patients with prostate cancer include a whole-blood control to distinguish CHIP from prostate cancer variants.

## Notes

Supplement. **eFigure.** All Variants Detected in Plasma Cell-Free DNA From 69 Men With Advanced Prostate Cancer. Each column represents 1 unique patient sorted by age.
