# Supplementary material for: Association of Clonal Hematopoiesis in DNA Repair Genes With Prostate Cancer Plasma Cell-free DNA Testing Interference
Source: JAMA Oncol. 2020 Nov 5;7(1):107–10. doi: 10.1001/jamaoncol.2020.5161 (PMC7645740; doi:10.1001/jamaoncol.2020.5161)
Supplement: Supplement. — eFigure. All Variants Detected in Plasma Cell-Free DNA From 69 Men With Advanced Prostate Cancer. Each column represents 1 unique patient sorted by age. [file jamaoncol-e205161-s001.pdf]

Supplemental Online Content

Jensen K, Konnick EQ, Schweizer MT, et al. Association of clonal hematopoiesis in DNA repair genes with prostate cancer plasma cell-free DNA testing interference. *JAMA Oncol*. Published online November 5, 2020. doi:10.1001/jamaoncol.2020.5161

eFigure. All Variations Detected in Plasma cell-free DNA from 69 men with Advanced Prostate Cancer

This supplemental material has been provided by the authors to give readers additional information about their work.

© 2020 Jensen K, et al. *JAMA Oncol*.

eFigure 1: All Variations Detected in Plasma cell-free DNA from 69 men with Advanced Prostate Cancer

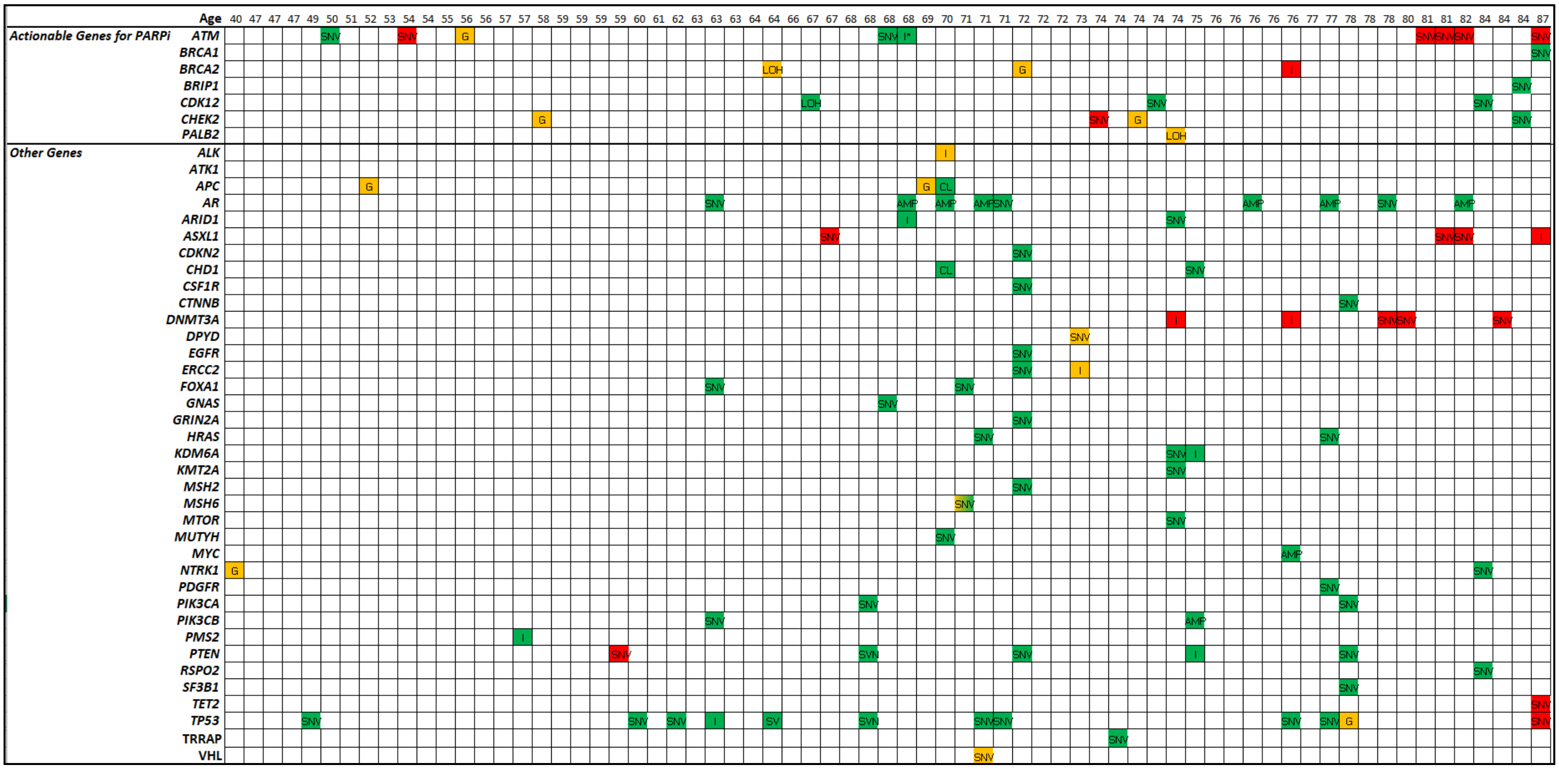

CHIP Interference Somatic	SNV: Single Nucleotide Variant
Non-CHIP Inteference Somatic	SV: Structural Variant
Germline Mutation	I: Indel
	CL: Copy Loss
	AMP: Gene amplification
	G: Germline clinically significant mutation (any type)
	Mix: Multiple mutations of different type
	LOH: Loss of heterozygosity/and or second hit
	AMP: Amplification or duplication
	*Deletion of -1 splice acceptor site
	**Germline with second hit

© 2020 Jensen K, et al. *JAMA Oncol*.
